# Diverse Genotypes of *Cryptosporidium* in Sheep in California, USA

**DOI:** 10.3390/pathogens11091023

**Published:** 2022-09-08

**Authors:** Xunde Li, Tamara Vodovoza, Edward R. Atwill

**Affiliations:** Department of Population Health and Reproduction, School of Veterinary Medicine, University of California, Davis, CA 95616, USA

**Keywords:** *Cryptosporidium*, genotype, sheep, zoonotic, *C. bovis*, *C. ubiquitum*, *C. xiaoi*

## Abstract

*Cryptosporidium* spp. is a parasite that can infect a wide variety of vertebrate species. The parasite has been detected in sheep worldwide with diverse species and genotypes of various levels of zoonotic potential and public health concern. The purpose of this study was to determine the distribution of genotypes of *Cryptosporidium* in sheep in California, USA. Microscopic positive samples from individual sheep from central and northern California ranches were genotyped by sequencing a fragment of the 18S rRNA gene and BLAST analysis. Eighty-eight (63.8%) of the microscopic positive samples were genotyped, and multiple genotypes of *Cryptosporidium* were identified from sheep in the enrolled ranches. Approximately 89% of isolates (n = 78) were *C. xiaoi* or *C. bovis*, 10% of isolates (n = 9) were *C. ubiquitum*, and 1% of isolates (n = 1) were *C. parvum*. The *C. parvum* and *C. ubiquitum* isolates were detected only from lambs and limited to four farms. Given that the majority of *Cryptosporidium* species (i.e., *C. xiaoi* and *C. bovis*) were of minor zoonotic concern, the results of this study suggest that sheep are not a reservoir of major zoonotic *Cryptosporidium* in California ranches.

## 1. Introduction

*Cryptosporidium* spp. parasites virtually infect all vertebrate animals, including humans, livestock species, companion animals, and a wide range of mammalian wildlife [1,2]. Among the nearly forty named species of *Cryptosporidium* [3], the majority of species are host-specific with an additional subset of zoonotic species and genotypes that are infectious to humans [4,5]. *Cryptosporidium* spp. that are considered zoonotic in alphabetical order include (major vertebrate host in parenthesis): *C. andersoni* (cattle), *C. bovis* (cattle), *C. canis* (dogs), *C. cuniculus* (rabbits), *C. erinacei* (tree squirrels), *C. fayeri* (kangaroo), *C. felis* (cats), *C. meleagridis* (turkeys), *C. muris* (mice), *C. parvum* (cattle), *C. scrofarum* (pigs), *C. suis* (pigs), *C. tyzzeri* (mice), *C. ubiquitum* (cattle), and *C. xiaoi* (sheep and goats). In addition, *Cryptosporidium* spp. chipmunk genotype I (chipmunk), horse genotype (horse), mink genotype (mink), and skunk genotype (skunk) have also been associated with human infections [4]. Among these zoonotic species and genotypes, *C. hominis* and *C. parvum* are responsible for the majority of human infections [5,6] as well as the majority of waterborne outbreaks in human communities [7]; therefore, these two species are considered major zoonotic species of public health concern. Livestock species infected with zoonotic *Cryptosporidium* species and genotypes are considered a public health risk due to the possibility of transmitting infective oocysts to humans through direct contact [8] or by contaminating sources of drinking or recreational water leading to human waterborne cryptosporidiosis [9,10].

*Cryptosporidium* infections in sheep have been reported globally from numerous countries [11]. The most common *Cryptosporidium* species reported in sheep are *C. ubiquitum*, *C. xiaoi*, and *C. parvum* [12]. However, infections with other species such as *C. andersoni*, *C. baileyi*, *C. bovis*, *C. canis*, *C. fayeri*, *C. hominis*, *C. ryanae*, *C. scrofarum*, and *C. suis* have also been reported in sheep [13,14,15]. Sheep infections with different *Cryptosporidium* species present a wide range of risks to public health. For example, because of the high load of fecal shedding of oocysts in infected sheep [16], when *C. parvum* or *C. hominis* dominates the sheep infections on a farm, it generates higher zoonotic risks to farmworkers and to environmental matrices, such as drinking water during conditions of rainfall and pasture runoff.

In the United States, previous work has indicated that *C. ubiquitum* is the dominant species infecting sheep in the state of Maryland on the east coast of the US, followed by *C. xiaoi* and *C. parvum* [17]. California, which is located on the west coast of the US, is a region of major livestock production including sheep. California has nearly 4000 sheep operations and over 555,000 sheep and lambs, ranking second largest in the US [18]. We previously completed an epidemiological study of the prevalence and intensity of fecal shedding of *Cryptosporidium* oocysts in sheep in California [16]. Using archived DNA samples from microscopic positive samples, the objective of the current work was to determine the distribution of zoonotic versus non-zoonotic *Cryptosporidium* species in this statewide survey of California sheep ranches.

## 2. Results

### 2.1. Genotypes of Cryptosporidium in Sheep in California

Among the 138 microscopic positive samples across all sheep ranches, 88 (63.8%) samples from infected individual animals were successfully genotyped by sequencing a fragment of the 18s rRNA gene. The alignment of the 88 sequences resulted in four genogroups of *Cryptosporidium* in sheep in California. Except for genogroup 1, which contained only one isolate, sequences in genogroups 2, 3, and 4 were composed of multiple variants (i.e., a, b, c, d, e, and f) due to several nucleotide differences between the sequences. Genogroup 1 contained one isolate; genogroup 2 contained nine isolates; genogroup 3 contained 34 isolates; and genogroup 4 contained 44 isolates (Table 1). To avoid redundancy of submitting identical sequences for each variant, fifteen sequences were selected to represent these four genogroups and within-genogroup variants and were deposited into GenBank with accession numbers ON245368–ON245383.

BLAST analysis indicated that the 1 isolate in genogroup 1 was 100% identical to *C. parvum* isolates in GenBank; the 9 isolates in 4 variants (a–d) of genogroup 2 were 99.63–100% identical to *C. ubiquitum*; the 34 isolates in 6 variants (a–f) of genogroup 3 were 99.49–100% identical to *C. xiaoi*; and the 44 isolates in 5 variants (a–e) of genogroup 4 were 99.62–100% identical to both *C. xiaoi* and *C. bovis* (Table 1). To summarize, 38.6% (34/88) of *Cryptosporidium* spp. in enrolled California sheep ranches were sequenced as *C. xiaoi*, 50% (44/88) were *C. bovis* or *C. xiaoi*, 10% (9/88) were *C. ubiquitum*, and only 1.1% (1/88) were *C. parvum*.

### 2.2. Distribution of Cryptosporidium by Sheep Age, Breed, Fecal Characteristics, and Ranch Location

Approximately 93% (82/88) of the genotyped *Cryptosporidium* isolates were from lambs. Among these lamb isolates, only one (2%) was *C. parvum* and nine (10%) were *C. ubiquitum*; the remaining 88% (72/82) of *Cryptosporidium* isolates were *C. xiaoi* (i.e., genogroup 3) or *C. bovis*/*C. xiaoi* (i.e., genogroup 4). Only one *Cryptosporidium* isolate was from a yearling ewe and was identified as *C. xiaoi*-c; the remaining five isolates were from ewes and were identified as either *C. xiaoi* or *C. bovis*. Because none of the genotyped samples were from diarrheic sheep, no association was found between the *Cryptosporidium* species and fecal characteristics (Table 2). Stratified by sheep breed, the only *C. parvum* isolate was detected from Dorper; the nine isolates of *C. ubiquitum* were found in Capay Red (n = 3), Suffolk (n = 2), and mixed breeds (n = 4) (Table 3). *C. xiaoi* was distributed among Dorset, Rambouillet, Suffolk, Targhee, and mixed breeds, while *C. xiaoi*/*bovis* was distributed among Capay Red, Dorper, Hampshire, Rambouillet, Suffolk, and mixed breeds (Table 3).

The single *C. parvum* isolate was detected from ranch No. 1 in Sonoma County in northern California. The nine isolates of *C. ubiquitum* were distributed across four ranches (No. 5, 6, 7, and 11) located in two counties in northern California. All *Cryptosporidium* isolates in sheep from other farms were either *C. xiaoi* or *C. bovis* (Table 4).

### 2.3. Phylogenetic Relationships between C. bovis, C. ubiquitum, and C. xiaoi from California and Other Geographical Locations

**Figure 1:**
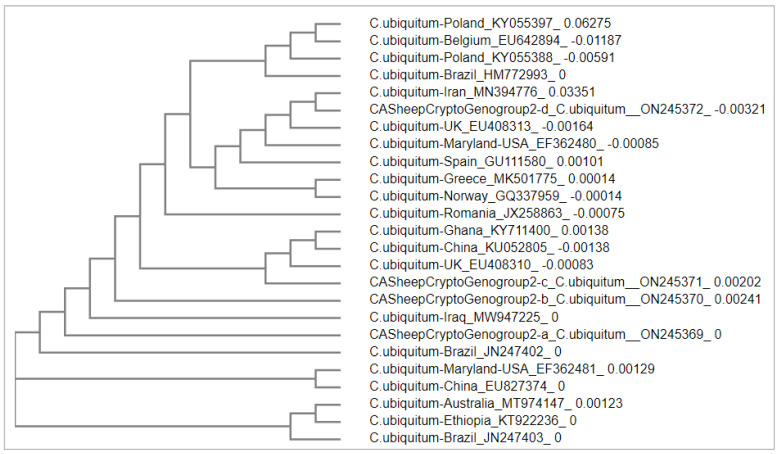
Phylogenetic relationships between *C. ubiquitum* from California sheep and a collection of representative *C. ubiquitum* isolates from sheep and goats from other worldwide locations. IDs of isolates start with the name of species or genotypes, followed by location and GenBank accession number.

The phylogenetic relationships between *C. ubiquitum* from California sheep and *C. ubiquitum* strains from other geographical locations are shown in Figure 1. The Californian *C. ubiquitum* (genogroup-a) is close to the strain isolated from Iraq; the genogroup-b and c formed a clade with strains from the UK, China, and Ghana; and the genogroup-d formed another clade with strains from Iran, the UK, Maryland, and Spain (Figure 1). These phylogenetic results indicate that variant strains of *C. ubiquitum* are widely distributed across diverse geographical locations.

**Figure 2:**
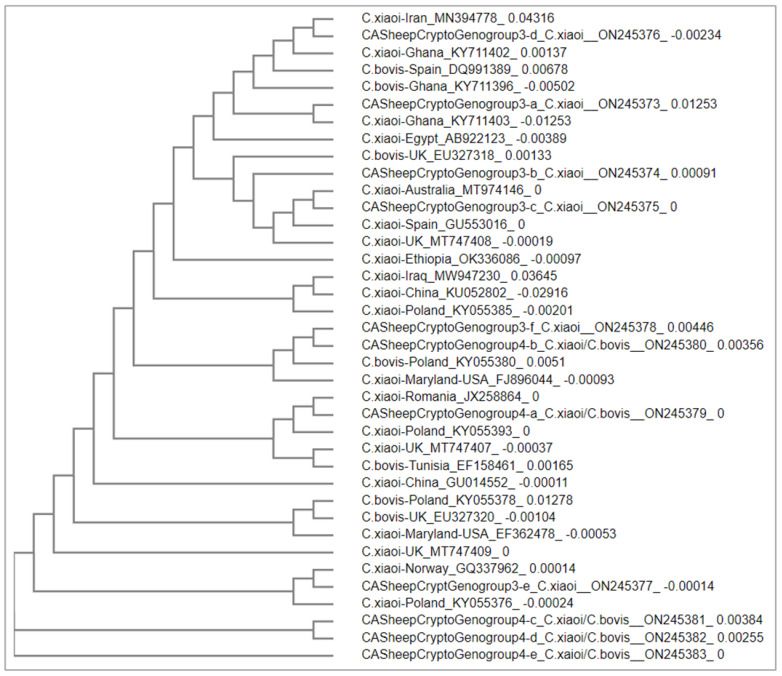
Phylogenetic relationships between *C. bovis*/*C. xiaoi* and *C. xiaoi* from California sheep and a collection of representative *C. bovis* and *C. xiaoi* isolates from sheep and goats from other worldwide locations. IDs of isolates start with the name of species or genotypes, followed by location and GenBank accession number.

*C. xiaoi* (genogroup 3 a–f) and *C. xiaoi*/*C. bovis* (genogroup 4 a–d) from sheep in California formed multiple clades with strains of *C. bovis* and *C. xiaoi* from sheep from various worldwide locations (Figure 2). *C. xiaoi* strains (a, b, c, and d) from California are in a clade with *C. xiaoi* and *C. bovis* from several countries, including Australia, Egypt, Ethiopia, Ghana, Spain, and the UK; *C. xiaoi*-e formed a clade with strains of *C. xiaoi* from Norway and Poland; and *C. xiaoi*-f formed a clade with *C. xiaoi*/*C. bovis* (genogroup 4 b) from California and *C. xiaoi* strains from China, Iraq, and Poland. *C. xiaoi*/*C. bovis* strains (genogroup 4 c, d, and e) are closely related to the clade of *C. xiaoi* from California, Norway, and Poland. *C. xiaoi*/*C. bovis* strains (genogroup 4 a) are in a clade with stains of *C. xiaoi* from Poland and Romania. The results indicate that (1) the *C. xiaoi*/*C.bovis* strains (genogroup 4) from California sheep are more likely related to *C. xiaoi,* and (2) various strains exist in *C. xiaoi* that are distributed across geographical locations.

## 3. Discussion

Given that the sequencing of the 18S rRNA gene is generally the most common method for the genotyping and speciation of *Cryptosporidium* spp. [6], the present study focused on the 18S rRNA sequences to compare *Cryptosporidium* from sheep throughout California with *Cryptosporidium* sequences in GenBank. Using the nucleotide BLAST’s default setting of targeting 100 sequences, genogroup 1 was 100% identical to 100 sequences of *C. parvum*; variants of genogroup 2 were 99.63–100% identical to 8 to 57 sequences of *C. ubiquitum*; variants of genogroup 3 were 99.49–100% identical to 3 to 7 sequences of *C. xiaoi* in GenBank. Because of the high sequence similarity, it is highly likely that the single isolate of genogroup 1 is *C. parvum*, the 9 isolates of genogroup 2 are *C. ubiquitum*, and the 34 isolates in genogroup 3 are *C. xiaoi*. For genogroup 4, given that the isolates with maximum sequence similarity were equivalent for both *C. xiaoi* and *C. bovis* from sheep and goats (Table 1), it is difficult to determine the species of *Cryptosporidium* for these 44 isolates in genogroup 4; they could be either *C. xiaoi* or *C. bovis*.

This confusion over which species of *Cryptosporidium* is present in a single fecal sample may also be the result of a mixed infection with more than one *Cryptosporidium* species in sheep; for example, *C. bovis* and *C. ubiquitum* mixed infection was observed in sheep in the UK [19], and *C. parvum* and *C. xiaoi* mixed infections were observed in sheep in Australia [20]. However, because the sequences were identical to more isolates of *C. xiaoi* than *C. bovis*, the genogroup 4 isolates could be more related to *C. xioai*. This assertion is supported by the phylogenetic analysis because genogroup 4 isolates were in clades closer to *C. xiaoi* than *C. bovis* (Figure 2). In summary, the combination of BLAST and phylogenetic analyses allowed us to identify *Cryptosporidium* species in sheep in California. Our results agree with previous reports that *C. xiaoi*, *C. ubiquitum*, and *C. parvum* are the most common *Cryptosporidium* species infecting sheep.

The distribution of the common *Cryptosporidium* species infecting sheep, namely, *C. xiaoi*, *C. ubiquitum,* and *C. parvum*, varies by worldwide geographical location [12]. *C. xiaoi* was the most common species in sheep in Egypt [21]; Ghana [22]; Tunisia [23]; Tanzania [24]; and Poland [25]. *C. ubiquitum* was the most common species in sheep/goat in Belgium [26]; Norway [27]; Brazil [28]; and Ethiopia [29]. *C. parvum* was found to be most common species in sheep in Spain [30,31,32,33]; Portugal [34]; Romania [35]; Italy [36]); Greece [37]; Zambia [38]); and Ireland [14]. In Australia, while two studies reported *C. xiaoi* as the most common species [20,39], a different pair of studies reported *C. ubiquitum* as most common species [13,40]. Another study found *C. parvum* as the most common species [41]. In the United Kingdom, similar contradictions occurred: one study found *C. xiaoi* was the most common species [42], while another study found *C. ubiquitum* as the most common species [19], and other studies reported *C. parvum* as the most common species [43,44,45]. Similarly, in China, some studies reported *C. xiaoi* as most common species [11,46,47], while other studies reported *C. ubiquitum* as most common species [48]. In the United States, a study reported *C. ubiquitum* as the dominant species followed by *C. xiaoi* and *C. parvum* in sheep in the state of Maryland [17] on the east coast.

In addition to geographical locations, the distribution of *Cryptosporidium* species in sheep can also vary by farm, sheep age, and season [11]. In our study, based on genotyping of >60% (88/138) of all the microscopic positive samples, nearly 90% (78/88) of *Cryptosporidium* from the California sheep were identified as *C. xiaoi* or *C. bovis*. *C. ubiquitum* comprised only 10% (9/88) of these isolates and *C. parvum* comprised only 1% (1/88). Given that *C. xiaoi*, *C. bovis*, and *C. ubiquitum* are of minor zoonotic concern due to few human cases being attributable to these species, our results indicate that sheep in California ranches are not a major reservoir of major zoonotic *Cryptosporidium* of public health concern. Our findings are in agreement with the reports of *Cryptosporidium* in sheep in Western Australia [13], which were also not a major reservoir of major zoonotic *Cryptosporidium*, based on the observation that the majority of genotyped *Cryptosporidium* from sheep were *C. ubiquitum,* which is not commonly found in humans. These findings suggest that sheep-derived *Cryptosporidium* might have been overestimated in the past as a significant cause of waterborne human cryptosporidiosis.

The single *C. parvum* isolate and all the isolates of *C. ubiquitum* were detected in lambs (Table 2). This could be due to the majority of the microscopic positive samples being from lambs (87.7% or 121/138); subsequently, the majority genotyped isolates were from lambs (93.2% or 82/88), in part due to lambs being more susceptible than yearlings or ewes to zoonotic infections with *C. parvum* and *C. ubiquitum*. In our previous work, we found a higher prevalence and higher intensity of oocyst shedding in lambs compared to yearlings and ewes; in addition, contact with cattle increased fecal oocyst shedding significantly [16]. Beneficial management practices, such as avoiding contact between sheep and cattle, and accessing surface water as drinking water, may help reduce the transmission of zoonotic *Cryptosporidium* species within and between livestock species.

Using existing knowledge of *Cryptosporidium* species of different zoonotic potential, this study assessed the zoonotic risks of *Cryptosporidium* from sheep in California. The findings of our studies suggest that diverse *Cryptosporidium* species are prevalent in different ages and breeds of sheep on California ranches, and that the majority of cryptosporidial species are not of significant public health concern. This work also contributes to the research of species and genotypes of *Cryptosporidium* infection in sheep worldwide.

## 4. Materials and Methods

### 4.1. Sample Collection

**Figure 3:**
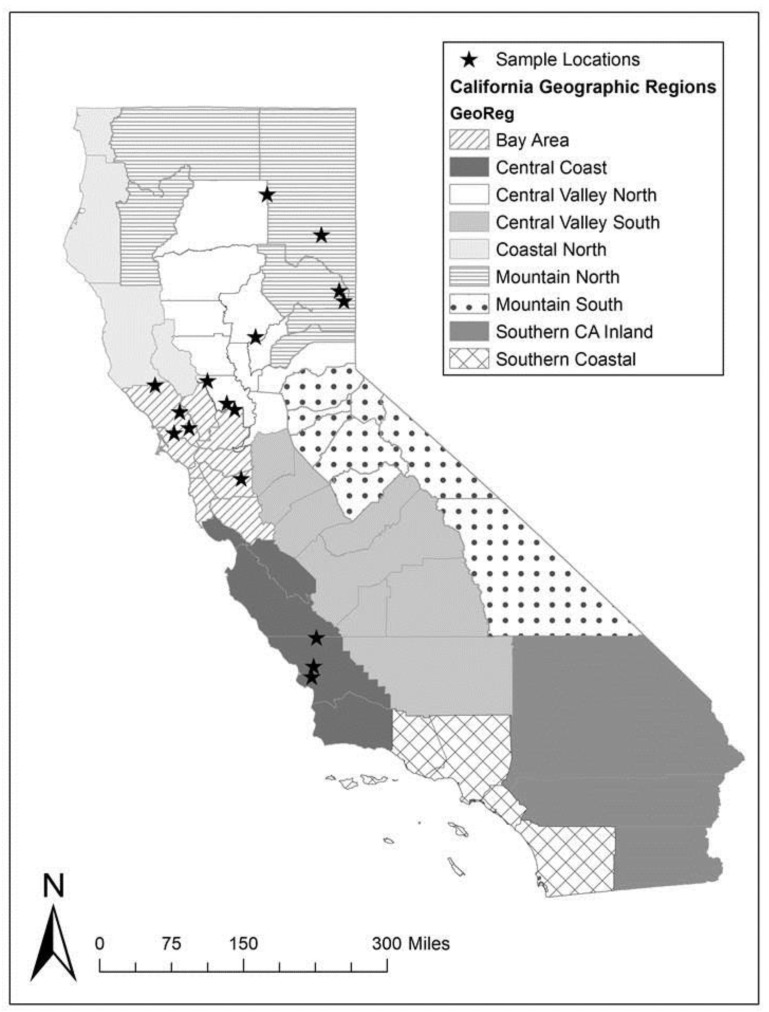
Sheep ranches in central and northern California enrolled in the study (n = 16) for sample collection.

An epidemiological study was conducted to investigate the prevalence of *Cryptosporidium* and intensity of fecal shedding of oocysts in sheep, and to identify risk factors for sheep infection in California, USA [16]. Through collaborations with livestock and natural resource advisors of the University of California Cooperative Extension, 16 sheep ranches located in Northern and Central California (Figure 3) were enrolled in this study based on voluntarily participation. Four ranches were located in the Mountain North region, four in the Central Valley North region, five in the San Francisco Bay Area, and three in the Central Coast region (Figure 3). A total of 798 fecal samples from 372 adult ewes, 31 yearlings, and 395 lambs were collected and tested for *Cryptosporidium* spp. We found that the overall prevalence of *Cryptosporidium* in California sheep was 17.3% (138/798), with access to surface sources of drinking water and contact with cattle being significantly associated with a higher risk of oocyst shedding in sheep of all ages [16]. Using archived DNA samples from this epidemiological study, the objective of the current work was to determine the genotypes of *Cryptosporidium* in sheep in California, USA.

### 4.2. DNA Extraction, PCR, and Sequencing

All fecal samples that were microscopic positive of *Cryptosporidium* oocysts were subjected to genotyping of *Cryptosporidium*. A 0.2 g of fresh feces was exposed to 5 cycles of freeze (−80 °C) and thaw (+70 °C), and then used for DNA extraction by using the DNA Stool Mini Kit (Qiagen, Hilden, Germany) according to the manufacturer’s instructions. All DNA samples were stored at −20 °C until further analysis. A nested PCR was performed on DNA samples using primers and reaction conditions amplifying an ~830 bp fragment of the 18S rRNA gene according to methods previously described [49,50]. A DNA template of *C. parvum* isolated from calves from a local dairy farm was used as a positive control, and a negative control without DNA template was included. PCR products were verified by electrophoresis in 2% agarose gel stained with ethidium bromide. Products of the secondary PCR were purified using Qiaquick spin columns (Qiagen) and sequenced at the UC Davis DNA Sequencing Facility using an ABI 3730 capillary electrophoresis genetic analyzer (Applied Biosystems Inc., Foster City, CA, USA). Primers of the secondary PCR were used for sequencing in both forward and reverse directions. Consensus sequences were generated from the forward and reverse sequences of each isolate using Vector NTI Advanced 11 software (Invitrogen Corporation, Carlsbad, CA, USA).

### 4.3. BLAST Analysis

To compare *Cryptosporidium* spp. isolates with existing reference species and genotypes of *Cryptosporidium* in GenBank, selected representative sequences of each genogroup were aligned with other *Cryptosporidium* sequences in GenBank using the NCBI’s online nucleotide basic local alignment search tool (BLAST). The BLAST analysis was optimized for highly similar sequences using default algorithm parameters and 100 maximum targeting sequences (6 April 2022, as last day accessed).

The rationale for conducting this BLAST analysis was that comparative genotyping is commonly used to broadly characterize the zoonotic or human-infection risk for a novel isolate of *Cryptosporidium*. For example, if the DNA sequence for a reasonably long section of the 18S rRNA gene from a *Cryptosporidium* isolate is either highly related (≥99.5%) or has 100% sequence homogeneity to a known zoonotic species or genotype, the isolate is typically considered to be zoonotic and infectious to humans. In contrast, if the DNA sequence for an isolate is not highly related to any known zoonotic species or genotypes of this parasite, it is generally considered not zoonotic. Although this decision process is not perfect, it is a current convention used by many researchers and regulatory agencies around the world to assign zoonotic disease risk of an isolate of *Cryptosporidium* found either in water, food, or animals.

### 4.4. Phylogenetic Analysis

Because of the diverse genotypes observed of *C. bovis/C. xiaoi* and *C. xioai* in sheep in California, we conducted a phylogenetic analysis to compare *C. bovis/C. xiaoi* and *C. xiaoi* from our study to *C. bovis and C. xiaoi* from sheep worldwide. Similarly, a phylogenetic analysis was conducted to compare *C. ubiquitum* from our study to *C. ubiquitum* from sheep worldwide. Sequence alignments were conducted using the online ‘Multiple Sequence Alignment’ tool at Clustal Omega (https://www.ebi.ac.uk/Tools/msa/clustalo/ (accessed on 6 May 2022)). Phylogenetic trees were constructed using the online ‘Simple Phylogeny’ tool (https://www.ebi.ac.uk/Tools/phylogeny/simple_phylogeny/ (accessed on 10 May 2022)) using the neighbor-joining method. Depending on the availability of sequences of *Cryptosporidium* from sheep in GenBank, reference sequences for the phylogenetic analyses were selected based on: (1) sequences of the 18s rRNA genes; (2) sequences of *C. bovis*, *C. ubiquitum*, and *C. xiaoi* from sheep/goat; (3) sequences representative of different geographical locations; and (4) sequence length (longer sequences available for each species, i.e., ~ 500 bp or longer) [51,52]. Information of *Cryptosporidium* species, locations, and GenBank accession numbers of selected sequences is available in Figure 1 and Figure 2.

## 5. Conclusions

The results of our study demonstrate that *C. xiaoi* was the dominant *Cryptosporidium* species isolated from sheep in California, which indicates that California sheep do not appear to be a major reservoir of zoonotic *Cryptosporidium* species of major public health concern in California ranches (i.e., not a major source of *C. parvum* or *C. hominis*). The findings of this work and our previous studies suggest that managing lamb health, avoiding contact with cattle, and using secure sources of drinking water for sheep may help to reduce the shedding of zoonotic *Cryptosporidium* in sheep in California ranches. Future studies are warranted to further investigate the geographical distributions and epidemiology of *Cryptosporidium* species in small ruminants.

## Figures and Tables

**Table 1:** Comparison of *Cryptosporidium* spp. from sheep in California with *Cryptosporidium* species and genotypes in GenBank by BLAST analysis.

*Cryptosporidium* Genotypes in Sheep in California			Highly Similar Sequences in GenBank (Last Access on 6 April 2022)		
*Cryptosporidium* Genogroup (No. of Samples)	Variant (No. of Samples)	GenBank Accession No.	*Cryptosporidium* Species and Host	Representative GenBank Accession No. *	Maximum Percent Identical (%)
CA sheep *Cryptosporidium* genogroup 1 (1)	a (1)	ON245368	*C. parvum*, goat	MT043934	100
CA sheep *Cryptosporidium* genogroup 2 (9)	a (5)	ON245369	*C. ubiquitum*, sheep	MH794165	100
	b (1)	ON245370	*C. ubiquitum*, sheep	MH794165	99.75
	c (1)	ON245371	*C. ubiquitum*, sheep	MH794165	99.63
	d (2)	ON245372	*C. ubiquitum*, Bactrian camels	MH442993	100
CA sheep *Cryptosporidium* genogroup 3 (34)	a (1)	ON245373	*C. xiaoi*, goat	MG602953	99.49
	b (1)	ON245374	*C. xiaoi*, goat	MG602953	99.87
	c (28)	ON245375	*C. xiaoi*, goat	MG602953	100
	d (1)	ON245376	*C. xiaoi*, goat	MG602953	99.62
	e (2)	ON245377	*C. xiaoi*, sheep	GU014553	100
	f (1)	ON245378	*C. xiaoi*, goat	MG602953	99.62
CA sheep *Cryptosporidium* genogroup 4 (44)	a (9)	ON245379	*C. xiaoi*, sheep *C. bovis*, sheep	MH049731 FJ608600	100 100
	b (1)	ON245380	*C. xiaoi*, goat *C. bovis*, sheep	MG602953 EU408315	99.73 99.73
	c (1)	ON245381	*C. xiaoi*, goat *C. bovis*, sheep	KT235699 EU827362	99.62 99.62
	d (1)	ON245382	*C. xiaoi*, goat *C. bovis*, sheep	KT235699 EU827362	99.75 99.75
	e (32)	ON245383	*C. xiaoi*, goat *C. bovis*, sheep	KT235699 EU827362	100 100

* To avoid redundancy, only one isolate was selected to represent maximal percent identical sequences. Genogroup 1 was 100% identical to 100 sequences of *C. parvum*; genogroup 2 isolates were 99.63–100% identical to 8–57 sequences of *C. ubiquitum*; genogroup 3 isolates were 99.49–100% identical to 3–7 sequences of *C. xiaoi*; genogroup 4 isolates were 99.62–100% identical to 7–11 sequences of *C. xiaoi* and 1–3 sequences of *C. bovis*.

**Table 2:** Distribution of *Cryptosporidium* genotypes in California sheep, stratified by age groups and fecal characteristics.

Age Group	Fecal Characteristics	No./No. Samples Genotyped	*Cryptosporidium* Genotype Group	Number of Samples
Lamb	Pellet	47/82	*C. parvum*	1
			*C. ubiquitum*-a	2
			*C. ubiquitum*-b	1
			*C. ubiquitum*-c	1
			*C. ubiquitum*-d	2
			*C. xiaoi*-a	1
			*C. xiaoi*-b	1
			*C. xiaoi*-c	9
			*C. xiaoi*-f	1
			*C. xiaoi/C. bovis*-a	7
			*C. xiaoi/C. bovis*-c	1
			*C. xiaoi*/*C. bovis*-e	20
	Pasty	35/82	*C. ubiquitum*-a	3
			*C. xiaoi*-c	16
			*C. xiaoi*-d	1
			*C. xiaoi*-e	2
			*C. xiaoi*/*C. bovis*-a	2
			*C. xiaoi*/*C. bovis*-d	1
			*C. xiaoi*/*C. bovis*-e	10
	Diarrhea	0/82		
Yearling	Pellet	0/1		
	Pasty	1/1	*C. xiaoi*-c	1
	Diarrhea	0/1		
Ewe	Pellet	2/5	*C. xiaoi*-c	1
			*C. xiaoi/C. bovis*-b	1
	Pasty	3/5	*C. xiaoi*-c	1
			*C. xiaoi/C. bovis*-e	2
	Diarrhea	0/5		

**Table 3:** Distribution of *Cryptosporidium* genotypes in California sheep, stratified by sheep breed.

Breed Name	No. of Sheep	Genotype	No. of Genotype
Capay Red	11	*C. xiaoi*/*bovis*-a	7
		*C. xiaoi*/*bovis*-b	1
		*C. ubiquitum*-c	1
		*C. ubiquitum*-d	2
Dorper	6	*C. parvum*	1
		*C. xiaoi*/*bovis*-e	5
Dorset	13	*C. xiaoi*-a	1
		*C. xiaoi*-c	4
		*C. xiaoi*/*bovis*-c	1
		*C. xiaoi*/*bovis*-e	7
Hampshire	7	*C. xiaoi*/*bovis*-e	7
Rambouillet	5	*C. xiaoi*-c	2
		*C. xiaoi*/*bovis*-e	3
Suffolk	24	*C. xiaoi*-c	15
		*C. xiaoi*-d	1
		*C. xiaoi*-f	1
		*C. xiaoi*/*bovis*-a	2
		*C. xiaoi*/*bovis*-d	1
		*C. xiaoi*/*bovis*-e	2
		*C. ubiquitum*-a	2
Targhee	5	*C. xiaoi*-b	1
		*C. xiaoi*-c	4
Mix *	17	*C. xiaoi*-c	3
		*C. xiaoi*-e	2
		*C. xiaoi*/*bovis*-e	8
		*C. ubiquitum*-a	3
		*C. ubiquitum*-b	1

* Mixed breeds of Dorper, Finnsheep, Targhee, Suffolk, Hampshire, or White face.

**Table 4:** Distribution of *Cryptosporidium* genotypes in California sheep, stratified by counties where the ranch was located.

Ranch ID	County	Prevalence of *Cryptosporidium*	No. Samples Genotyped/No. Positive Samples	*Cryptosporidium* Genotypes	Number of Samples
1	Sonoma	10.2% (5/49)	4/5	*C. parvum*	1
				*C. xiaoi*-c	2
				*C. xiaoi*/*C. bovis*-a	1
2	Yolo	21.6% (11/51)	11/11	*C. xiaoi*-c	1
				*C. xiaoi*/*C. bovis*-e	10
3	Yolo	16.0% (8/50)	7/8	*C. xiaoi*-c	3
				*C. xiaoi*-e	1
				*C. xiaoi*/*C. bovis*-e	3
4	Yolo	13.7% (7/51)	5/7	*C. xiaoi*-c	1
				*C. xiaoi*/*C. bovis*-e	4
5	Sonoma	32.0% (16/50)	9/16	*C. ubiquitum*-a	1
				*C. xiaoi*-c	5
				*C. xiaoi*/*C. bovis*-a	3
6	Sonoma	16.7% (8/48)	2/8	*C. ubiquitum*-a	1
				*C. ubiquitum*-c	1
7	Mendocino	25.5% (13/51)	5/13	*C. ubiquitum*-b	1
				*C. xiaoi*-a	1
				*C. xiaoi*-b	1
				*C. xiaoi*-c	1
				*C. xiaoi*-f	1
8	Plumas	19.2% (10/52)	7/10	*C. xiaoi*-c	3
				*C. xiaoi*/*C. bovis*-a	1
				*C. xiaoi*/*C. bovis*-e	3
9	Plumas	10.2% (5/49)	1/5	*C. xiaoi*/*C. bovis*-e	1
10	Lassen	13.0% (7/54)	6/7	*C. xiaoi*-c	2
				*C. xiaoi*/*C. bovis*-a	1
				*C. xiaoi*/*C. bovis*-e	3
11	Lassen	18.2% (10/55)	8/10	*C. ubiquitum*-a	3
				*C. ubiquitum*-d	2
				*C. xiaoi*-e	1
				*C. xiaoi*/*C. bovis*-a	1
				*C. xiaoi*/*C. bovis*-e	1
12	San Luis Obispo	14.5% (9/62)	5/9	*C. xiaoi*-c	4
				*C. xiaoi*/*C. bovis*-b	1
13	San Luis Obispo	14.5% (8/55)	6/8	*C. xiaoi*-c	4
				*C. xiaoi*/*C. bovis*-e	2
14	San Luis Obispo	26.7% (16/60)	10/16	*C. xiaoi*-c	2
				*C. xiaoi*-d	1
				*C. xiaoi*/*C. bovis*-a	2
				*C. xiaoi*/*C. bovis*-c	1
				*C. xiaoi*/*C. bovis*-e	4
15	Butte	16.1% (5/31)	2/5	*C. xiaoi*/*C. bovis*-d	1
				*C. xiaoi*/*C. bovis*-e	1
16	Contra Costa	0% (0/30)	0/0		

## Data Availability

The DNA sequences of *Cryptosporidium* from sheep in California and around the world are available at https://www.ncbi.nlm.nih.gov/nuccore, with the accession number of each sequence cited in the text of the article.
